# Ex vivo lung-organoid model for aberrant basaloid cell induction and activation

**DOI:** 10.1186/s41232-025-00396-z

**Published:** 2025-10-30

**Authors:** Bin Wu, Shigeyuki Shichino, Satoshi Ueha, Rina Matsukiyo, Yu Ishimura, Haru Ogiwara, Masaki Takasu, Shotaro Yamano, Yumi Umeda, Kouji Matsushima

**Affiliations:** 1 Division of Molecular Regulation of Inflammatory and Immune Diseases, Research Institute for Biomedical Science, Tokyo University of Science, Noda, Chiba, Japan; 2 ImmunoGeneTeqs, Inc., Kashiwa-shi, Chiba, Japan; 3 Center for One Medicine Innovative Translational Research (COMIT), Institute for Advanced Study, Gifu University, Gifu, Japan; 4 Graduate School of Medicine, Dentistry and Pharmaceutical Sciences, Okayama University, Okayama, Japan; 5 National Institute of Occupational Safety and Health, Japan Organization of Occupational Health and Safety, Fujisawa, Kanagawa Japan

**Keywords:** Three-dimensional lung organoid, Lung epithelial injury, Aberrant basaloid cells, Idiopathic pulmonary fibrosis

## Abstract

**Background:**

Pulmonary fibrosis (PF) is a severe lung disease characterized by the destruction of lung architecture resulting from chronic epithelial injury. The PF microenvironment induces PF-specific epithelial cells, such as aberrant basaloid cells (ABCs). However, limited experimental models capable of inducing and activating PF-specific epithelial cells hinder the understanding of their roles.

**Methods:**

To address the lack of experimental models, in this study, we developed an ex vivo murine lung-organoid model designed to induce and activate ABCs. The organoids were subjected to bleomycin (BLM) stimulation. Dose-dependent reductions in number and size, structural disorganization, and transcriptomic changes were assessed following stimulation. Single-cell RNA-sequencing (scRNA-seq) analysis was performed to identify ABC subsets. Cell–cell interaction analysis was also conducted.

**Results:**

Following BLM stimulation, the organoids displayed dose-dependent reductions in number and size, along with structural disorganization and transcriptomic changes that were similar to those observed in the in vivo murine fibrosis model. scRNA-seq analysis identified two ABC subsets: Krt5^low^ Tp63^low^ Krt17^+^ ABCs_1, found in patients with idiopathic pulmonary fibrosis (IPF), and Krt5^hi^ Tp63^hi^ Krt17^+^ ABCs_2, which have been observed in cultured tissues from patients with IPF but not in traditional murine models. BLM stimulation led to the induction of transforming growth factor beta (TGF-β2) expression in ABCs. Cell–cell interaction analysis suggested that BLM-damaged type 2 alveolar epithelial cells (AT2s) enhanced their direct and indirect interactions with ABCs_2 via ephrin-A signaling. In line with this observation, stimulation experiments of BLM-damaged organoids revealed that Ephrin A4 induced ABC cell differentiation-related gene expression changes, whereas Ephrin A3 enhanced epithelial proliferation-related gene expression changes and suppressed fibroblast activation-related gene expression changes.

**Conclusions:**

The developed organoid model serves as a novel platform for studying the roles and responses of PF-specific ABCs. This model may contribute to advancing the understanding of PF pathogenesis and facilitate the development of ABC-targeted therapies.

**Supplementary Information:**

The online version contains supplementary material available at 10.1186/s41232-025-00396-z.

## Background

The lungs are the central organs of the respiratory system and comprise alveoli and bronchioles. The alveoli are lined with alveolar epithelial cells (AECs), which play a key role in lung function and maintain normal alveolar structures. AECs mainly comprise self-renewing type 2 alveolar epithelial cells (AT2), which secrete surfactants and other proteins to aid in alveolar stretching and contraction, and type 1 alveolar epithelial cells (AT1), which contribute to gas exchange with the surrounding capillaries [1–3]. The bronchiole is lined with basal, secretory, and ciliated cells and plays a role in maintaining mucus barrier function. In normal lung repair processes, damaged epithelial cells are recovered; however, in chronic lung diseases such as idiopathic pulmonary fibrosis (IPF), these lung structures are destroyed, eventually leading to the loss of lung function [4]. Recently, single-cell RNA-seq (scRNA-seq) technologies that can identify the characteristics of each cell in an unsupervised manner have evolved, and IPF-specific abnormal epithelial cells have been identified based on atlas-scale scRNA-seq studies. One of these is the aberrant basaloid cell (ABC), which is characterized by the expression of the basal cell marker KRT17 along with mesenchymal cell markers (FN1), senescence markers, IPF-associated molecules, while lacking canonical AEC markers such as SFTPC and homeodomain-only protein X (HOPX), which are specifically surrounded by fibrotic foci in patients with IPF [5, 6]. However, to the best of our knowledge, the lack of an experimental model that can induce ABCs limits our understanding of their pathophysiological roles in lung fibrosis.

Organoid culture, which are three-dimensional cultures of organ progenitors, has been widely used to reproduce organ-like structures and multiple organ-specific cell-type features in vitro [7]. Organoids elucidate tissue repair and disease mechanisms by mimicking cell–cell and molecular interactions in real organs. Lung organoids, including alveolospheres and tracheospheres, can be generated from pluripotent embryonic stem cell (ESC)/induced pluripotent stem cell (iPSC)-derived lung epithelial cells or primary lung epithelial progenitor cells, such as AT2 and basal cells [7–10]. Single basal cells isolated from the trachea can grow into tracheospheres comprising a pseudostratified epithelium of basal and ciliated cells [11]. Co-cultures of primary AECs and lung fibroblasts create alveolospheres that represent the distal lung [12, 13], and Shiraishi et al. previously revealed that supplementation of lung fibroblast-derived factors could form alveolospheres only by AEC monoculture [14]. Recently, Suezawa et al. reported that bleomycin (BLM), an anticancer drug widely used to generate a murine lung fibrosis model, induces fibrotic responses in lung organoids generated from human iPSC-derived lung epithelial cells [15]. This report highlights the usefulness of lung-organoid models for analyzing lung injury and fibrotic responses in vitro. However, the absence of an ex vivo organoid model that can induce or activate ABCs, a hallmark of IPF, is a major barrier to elucidating the pathophysiological roles of abnormal epithelial cells in IPF.

In this study, we generated a mixture of murine alveolospheres and tracheospheres within a 3D Matrigel scaffold using primary murine lung epithelial cells and fibroblasts. BLM stimulation induced lung injury/fibrosis-associated changes in the organoids, and scRNA-seq analysis of the organoids revealed the emergence of two ABC-like subsets (ABCs_1 and ABCs_2), which were defined by the expression of *Krt17*, *Fn1*, and variable levels of *Krt5* and *Trp63*; however, they lacked canonical AECs markers such as *Sftpc* and *Hopx,* as observed in human IPF lungs. Furthermore, ABCs_2 expressed TGF-β2, a cytokine associated with ABC activation in IPF. In addition, cell–cell interaction analysis and stimulation experiments suggested that injured AT2 cells may directly interact with ABCs_2 via Ephrin A4 signaling, which may promote ABC differentiation. Collectively, this murine model could provide a convenient tool for studying the functions and properties of ABC-like cells in lung injury and fibrosis.

## Materials and methods

### Animal and study approvals

C57BL/6 and CAG-EGFP transgenic C57BL/6 mice were purchased from Sankyo Lab Service (Tokyo, Japan). All mice were maintained in specific pathogen-free facilities at the Tokyo University of Science and used for experiments at 8–10 weeks of age. All experiments were conducted with the approval of the Animal Experiment Committee of the Tokyo University of Science (S18029, S19024, and S20019) and in accordance with the laws and regulations of the Animal Experiment Manual of the Tokyo University of Science.

### Preparation of murine lung cells

Lung cell suspensions were prepared as previously described, with minor modifications [16]. Briefly, the mice were anesthetized with isoflurane, their lungs were perfused with phosphate-buffered saline (PBS; Nacalai Tesque, Kyoto, Japan), and the whole lungs were collected. Murine lung samples were minced into 0.5–mm^2^ pieces with a razor blade and enzymatically digested in solution [0.2 mg/mL collagenase (Fujifilm Wako Pure Chemicals, Tokyo, Japan), 0.96 mg/mL Dispase II (Roche, Basel, Switzerland), 20 kU/mL DNase I (Sigma-Aldrich, MO, USA) in preparation medium [RPMI-1640 medium (Nacalai Tesque) supplemented with 10 mM HEPES (Nacalai Tesque) and 10% fetal bovine serum (FBS)] at 37 °C for 30 min. After enzymatic digestion, the cells were agitated 10 times with an 18G syringe needle and then incubated at 37 ℃ for 20 min. The cell suspension was centrifuged at 500 × *g* for 5 min at 20 °C, the supernatant was removed, and the cell pellet was resuspended and agitated 10 times with a 21G syringe needle in 25% Percoll (GE Healthcare, IL, USA). Thereafter, 65% Percoll was under layered, centrifuged at 1000 × g for 20 min at 20 °C, the middle layer was collected, diluted in 8 mL of preparation medium, and centrifuged again at 500 × *g* for 8 min at 4 °C. The resulting pellet was resuspended in PBS with 0.5% bovine serum albumin (Nacalai Tesque) and filtered through a 40 µm cell strainer (Corning Falcon, AZ, USA).

### Magnetic separation and cell sorting

For murine lung fibroblast isolation, single-cell suspensions from the lungs of C57BL/6 J mice were stained with an antibody cocktail [biotin anti-mouse CD31 (2.5 μg/mL; Clone: 390, BioLegend, CA, USA), CD45 (2.5 μg/mL; clone: 30-F11, BioLegend), Ter-119 (2.5 μg/mL; clone: TER-119, BD Biosciences, NJ, USA), CD146 (2.5 μg/mL; clone:ME-9F1, BioLegend), and EpCAM (2.5 μg/mL; clone: G8.8, BioLegend) antibodies] for 20 min at 4 ℃, washed once with 10% FBS in PBS, and stained with a secondary antibody cocktail (APC anti-mouse CD140a (2 μg/mL; clone: APA5, BioLegend) and PE streptavidin (2.5 μg/mL, BioLegend)) for 20 min at 4 °C. After washing once with 10% FBS in PBS, lineage (CD31, CD45, CD146, Ter119, and EpCAM)^+^ cells were labeled using anti-phycoerythrin (PE) microbeads (Miltenyi Biotec, Bergisch Gladbach, Germany) for 15 min at 4 °C. After washing once with 10% FBS/PBS, lung fibroblasts were enriched by negative selection using LS columns (Miltenyi Biotech). For murine lung epithelial cell isolation, single-cell suspensions from the lungs of green fluorescent protein (GFP) mice were stained with an lineage antibody cocktail [biotin anti-mouse CD31 (2.5 μg/mL; clone: 390, BioLegend), CD45 (2.5 μg/mL; clone: 30-F11, BioLegend), Ter-119 (2.5 μg/mL; clone: TER-119, BD Biosciences), CD146 (2.5 μg/mL; clone: ME-9F1, BioLegend), and CD140a (2.5 μg/mL; clone: APA5, BioLegend) antibodies] for 20 min at 4 ℃, washed once with 10% FBS in PBS, and finally stained with a secondary antibody cocktail (APC-streptavidin (2.5 μg/mL BioLegend) and PE-Cy7 anti-mouse EpCAM (2 μg/mL; clone: G8.8, BioLegend)) for 20 min at 4 °C. After washing once with 10% FBS in PBS, lineage (CD31, CD45, CD146, Ter119, and CD140a^+^) cells were labeled using anti-APC microbeads (Miltenyi Biotec) for 20 min at 4 °C and washed once with 10% FBS/PBS. Epithelial cells were enriched by negative selection using LS columns (Miltenyi Biotech). The resulting cells were further purified by cell sorting (CD31^−^ CD45^−^ CD146^−^ Ter119^−^ EpCAM^−^ propidium iodide (PI)^−^ CD140a^✛^ cells as fibroblasts, and CD31^−^ CD45^−^ CD146^−^ Ter119^−^ PI^−^ GFP^+^ EpCAM^+^ cells as epithelial cells) using the BD FACSAria™ II or BD FACSAria™ III cell sorter (BD, NJ, USA).

### Preparation of murine lung organoids

Murine lung organoids were prepared by co-culturing lung fibroblasts and epithelial cells, as described previously [14]. Briefly, sort-purified murine lung fibroblasts and epithelial cells were centrifuged at 800 × *g* for 5 min at 4 °C, the supernatant was discarded, and cells were resuspended with MTEC Plus medium [DMEM/F-12 (Sigma-Aldrich) with 5% FBS supplemented with mouse epidermal cell growth factor (25 ng/mL; R&D Systems, MN, USA), pituitary extract (30 µg/mL; Sigma-Aldrich), insulin–transferrin–sodium selenite (5 µg/mL; Thermo Fisher Scientific), 1.5 mM l-glutamine, 100 U/mL penicillin, 100 μg/mL streptomycin, 0.03% (w/v) NaHCO_3_ (Gibco, Thermo Fisher Scientific), and cholera toxin (100 ng/mL; Sigma-Aldrich)], supplemented with retinoic acid (0.01 µM; Sigma-Aldrich) to a concentration of 4 × 10^6^ cells/mL (fibroblasts) and 2 × 10^5^ cells/mL (epithelial cells). Thereafter, 25 µL of lung fibroblasts and epithelial cell suspensions were mixed in a 1.5 mL tube and mixed with 50 µL Matrigel (Corning) on ice. Next, 90 µL of cell suspension was placed in a 24-well 0.4-µm Transwell clear insert (Falcon; BD Biosciences or Greiner Bio-One, Austria) and incubated for 30 min at 37 °C in a 5% CO_2_ incubator with humidified atmospheres for Matrigel polymerization. Finally, 500 µL of MTEC Plus medium supplemented with retinoic acid (0.01 µM; Sigma-Aldrich) and Y-27632 Rho-associated kinase inhibitor (10 µM; Fujifilm Wako Pure Chemicals, Tokyo, Japan) was added to each well and cultured at 37 °C in a 5% CO_2_ incubator with a humidified atmosphere. The culture medium (MTEC Plus) was replaced every 2 days. Retinoic acid and Y-27632 hydrochloride stocks were prepared and diluted with sterile water and stored at − 80 and − 20 °C, respectively, and were added just before use. Culture media containing retinoic acid were protected from light exposure.

### BLM stimulation of lung organoids

On day 8 post co-culture, lung organoids were stimulated with BLM by replacing the culture medium with MTEC Plus medium supplemented with 0, 0.01, 0.1, 0.3, and 1 µg/mL of BLM. After 2 days of stimulation, the BLM-containing medium was removed and replaced with the MTEC Plus medium.

### Imaging analysis

To analyze the lung organoid size and number, images of the entire Transwell stored in 24-well plates were captured on days 8, 10, and 14 post co-culture using a BZ-X800 fluorescence microscope (Keyence, Osaka, Japan) (basic imaging conditions: 4 × magnification, exposure time; GFP = 1/4 s and bright field = 1/4000 s). Organoids within the entire Transwell were automatically counted using GFP fluorescence images with hybrid cell counting software (Keyence). The size exclusion of the hybrid cell counting software was set to 1000 µm^2^_._ The size of each organoid was measured automatically using hybrid cell counting software.

### Immunofluorescence of lung organoids

For immunofluorescence (IF) staining of lung organoids, we prepared lung organoids (1:1 ratio of lung epithelial cells to lung fibroblasts) within a 3.5 mm glass-bottom dish (Matsunami, Osaka, Japan) instead of a Transwell chamber. On day 14 post co-culture, the medium was removed, and the organoids were washed once with 2 mL of PBS. Organoids were fixed with 2 mL of 4% paraformaldehyde (PFA)/PBS (Fujifilm Wako Pure Chemicals) for 1 h at room temperature and washed thrice with 2 mL of PBS. The fixed organoids were permeabilized with 2 mL of 0.5% TritonX-100 (Sigma-Aldrich) in PBS for 1 h at room temperature, washed twice with PBS, and blocked with 2 mL of 1% BSA/PBS in PBS (w/v) for 1 h at room temperature. Lung organoids were stained with primary antibodies (anti-Prosurfactant Protein C, 2 µg/mL; anti-Hopx, 0.4 µg/mL) overnight at 4 ℃, washed with PBS, and stained with the appropriate secondary antibodies (Alexa 555 plus donkey anti-rabbit IgG 2 µg/mL, Thermo Fisher Scientific; Alexa 647 donkey anti-mouse IgG, 2 µg/mL, Thermo Fisher Scientific) for 4 h at room temperature. After washing it thrice with PBS, the organoids were stained with DAPI/PBS (D1306; Thermo Fisher Scientific) for 30 min at room temperature and washed thrice with PBS. The organoids were covered with 250 µL of ProLong Diamond Antifade Mountant (Invitrogen, Thermo Fisher Scientific) and coverslips. Images were acquired using an SP-5 confocal microscope (Leica, Wetzlar, Germany) at × 20 magnification. The organoids were protected from light during all antibody-staining processes.

### Bulk transcriptome library preparation and sequencing

Bulk transcriptome libraries of lung organoid-derived cells were prepared as previously described, with some modifications [17]. Briefly, on day 14 post co-culture, lung organoids treated with BLM (0, 0.01, 0.1, and 1.0 µg/mL) were directly collected and resuspended in 500 µL cell lysis buffer [100 mM Tris–HCl pH 7.5, 1% (w/v) lithium dodecyl sulfate (Nacalai Tesque), 500 mM LiCl, 10 mM EDTA, and 5 mM dithiothreitol (Thermo Fisher Scientific)]. PolyA RNAs were isolated using Dynabeads M-270 streptavidin (Thermo Fisher Scientific) conjugated with biotin-3′ WTA-EcoP-dT25 according to the previous report, as some modifications [17]. To perform reverse transcription, the beads were suspended in 10 μL of RT mix [5 × Superscript IV buffer (Thermo Fisher Scientific), 1 mM dNTP (Roche), 5 mM dithiothreitol (Thermo Fisher Scientific), 1 M betaine (Sigma-Aldrich), 6 mM MgCl_2_, 1 U/µL RNaseIn Plus Rnase Inhibitor (Promega, Madison, WI, USA), 10 U/μL Superscript IV (Thermo Fisher Scientific)] and incubated for 5 min at 35 °C, 30 min at 42 °C, and immediately chilled on ice. The beads were washed once with B&W-T buffer [5 mM Tris–HCl (pH 8.0) (Nippon Gene, Tokyo, Japan), 1 M NaCl (Merck, NJ, USA), 1 mM EDTA (Nippon Gene), and 0.05% Tween-20 (Merck)] and once with 10 mM Tris–HCl pH 8.0. To digest free RT primers, the beads were suspended in 10 µL of Exo I mix [10 × Exo I buffer (New England Biolabs, MA, USA) and 2 U/µL Exonuclease I (New England Biolabs)], incubated for 30 min at 37 °C, and immediately chilled on ice. The beads were washed twice with a B&W-T buffer and once with 10 mM Tris–HCl (pH 8.0). To add polyC tails, beads were suspended in 10 µL of polyC tailing mix [10 × Thermopol buffer (New England Biolabs), 2 mM dCTP (Roche), 0.1 mM ddCTP (GE Healthcare), 1 mM CoCl_2_ (Roche), RNase H (Invitrogen, Thermo Fisher Scientific, 15 U/µL TdT enzyme (Roche)], incubated for 30 min at 37 °C, and immediately chilled on ice. The beads were washed once with a B&W-T buffer and then with 10 mM Tris–HCl (pH 8.0). To perform the 2nd strand synthesis reaction, beads were suspended in 10 μL of 2nd strand mix [1 × KAPA Hifi Hotstart ReadyMix (KAPA Biosystems, MA, USA), 0.4 µM of Illumina-i7-9G primer], and PCR was performed as follows: 3 min at 95 °C, 20 s at 98 °C, 16 cycles of [1 min at 47 °C and 2 min at 72 °C], followed by 5 min at 72 °C, and hold at 4 °C. The 1st whole-transcriptome amplification was performed by adding 15 µL of the first PCR mix [0.32 µM primer (illumina-i7), 0.48 µM primer (NH2-3’ WTA), and 1 × KAPA Hifi Hotstart ReadyMix] into the second strand synthesis reactions, and PCR was performed as follows: 3 min at 95 °C, 12 cycles of [20 s at 98 °C, 15 s at 65 °C, and 5 min at 72 °C], followed by 5 min at 72 °C, and held at 4 °C. The first PCR products were purified using AmPure XP beads (Beckman Coulter, CA, USA) at a 0.6:1 ratio of reagents to sample and eluted with 25 μL of nuclease-free water. Approximately 6.3 µL of the purified first PCR product was mixed with 8.7 μL of the second PCR mix [0.4 μM of primers (illumina-i7, NH2-3’ WTA), and 1 × KAPA Hifi Hotstart ReadyMix]. PCR was performed as follows: 3 min at 95 °C, five cycles of [20 s at 98 °C, 15 s at 65 °C, and 5 min at 72 °C], followed by 5 min at 72 °C, and held at 4 °C. The second PCR products were purified using AmPure XP beads at a 0.6:1 ratio of reagent to sample and eluted with 15 μL of 10 mM Tris–HCl pH 8.0. Sequencing library preparation was performed using the NEBNext Ultra II FS DNA Library Prep Kit for Illumina (New England Biolabs) according to the manufacturer’s instructions, with some modifications. Approximately 100 ng of the amplified cDNA library was subjected to fragmentation/end-repair/A-tailing as follows: 7 min at 32 °C, 30 min at 65 °C, and held at 4 °C. Thereafter, 1.25 μL of 1.5 µM Illumina adapter was used for adapter ligation. Ligated products were purified by double size selection with 0.4 × → 0.7 × (final 1.1 ×) AmPure XP beads and eluted with 10 µL of nuclease-free water. Barcoding PCR was performed with 22.5 µL of barcoding mix [7.5 µL of the resulting eluates, 1 µM primers (ILMN_[UDI]_i5 and ILMN_[UDI]_i7), and 1 × NEBNext Ultra II Q5 (New England Biolabs)], and PCR was performed as follows: 30 s at 98 °C, nine cycles of [10 s at 98 °C and 75 s at 65 °C], followed by 5 min at 65 °C, and hold at 4 °C. The resultant products were purified twice by double size selection with 0.5 × → 0.8 × (final 1.3 ×) AmPure XP beads and eluted with 12 µL of 10 mM Tris–HCl pH 8.0. The size distribution of the amplified products was analyzed using a MultiNA system (Shimadzu, Kyoto, Japan). The final transcriptome libraries were quantified using the KAPA Library Quantification Kit (KAPA Biosystems). Sequencing was performed using a NovaSeq S4 200 cycle kit v1.5 and NovaSeq 6000 sequencer (Illumina, San Diego, CA, USA) according to the manufacturer’s instructions (read 1 67 bp, read 2 140 bp). A 12% phiX spike-in was used for the sequencing. Raw sequencing data were deposited in the National Center for Biotechnology Information (NCBI) Gene Expression Omnibus database (GEO, http://www.ncbi.nlm.nih.gov/geo; accession number: GSE200299).

### Analysis of bulk transcriptome data

Quality filtering of the sequencing data was performed using Cutadpat-v2.10 [18]. The filtered reads were mapped to the reference RNA (GRCm38 release-101) using Bowtie2-2.4.2 [19] (parameters: -p 2 -L 16 –very-sensitive-local -N 1 -nofw -seed 656,565 -reorder), and the number of reads of each gene was counted. Transcriptome data were analyzed as previously reported [20]. Briefly, between-sample normalization was performed against raw count data using R version 3.5.1. (https://cran.r-project.org/) and the TCC package (EEE-E method) [21, 22]. Genes for which adjusted *p* < 0.05, fold change ≥ 2, and maximum expression ≥ 100 were identified as statistically significant differentially expressed genes (DEGs). Co-expressed gene modules among the DEGs were detected using the weighted co-expression network analysis package [23]. Functional analysis of gene module groups was performed using Cytoscape 3.7.2 with the ClueGO plugin (v2.5.7) [24, 25]. Significantly enriched Gene ontology (GO) terms [26] (GO biological processes, GO levels: 3–8) and Kyoto Encyclopedia of Genes and Genomes (KEGG, version 2020/8/5) pathway terms [27] in the gene module groups were explored and grouped. A term network was constructed based on the overlap of elements (χ score = 0.4). The leading terms within each group were defined as the most significantly enriched terms.

### scRNA-seq library preparation of lung organoids

Lung organoids embedded in Matrigel were incubated with Accutase solution (Sigma-Aldrich) at 37 °C for 20 min, followed by incubation with 0.25% trypsin–EDTA (Nacalai Tesque) at 37 °C for 10 min. Trypsin was inactivated using DMEM/F12 supplemented with 10% FBS, and the cells were resuspended in PBS supplemented with 0.01% BSA. Single-cell RNA-sequencing (scRNA-seq) library preparation was performed according to a previous report, with modifications [28]. The resulting single-cell suspension of lung organoids was stained with 50 μL of 2.5 μg/mL BD anti-mouse sample tag antibodies (anti-mouse sampletag 1–6 and 8 (anti-MHC class I), BD Biosciences) diluted with Cell Staining Buffer (BioLegend) for 30 min at 4 °C. After washing thrice with Cell Staining Buffer, the cells were stained with Calcein AM for 5 min at 37 °C, immediately chilled on ice, and filtered through a 40 µm cell strainer. Calcein AM^+^ PI^−^ live cells were counted using flow-count fluorospheres (Beckman coulter) and a CytoFLEX S flow cytometer (Beckman Coulter), and cell suspensions were pooled as their cell number were equal. scRNA-seq library preparation was performed as previously described, with modifications. The resulting 25,000 single cells were analyzed using a BD Rhapsody system with a BD Rhapsody Targeted & Abseq Reagent kit (BD Biosciences), following the manufacturer’s instructions. After reverse transcription, the resultant BD Rhapsody beads were treated with exonuclease I at 37 °C for 60 min at 1200 rpm on a Thermomixer C with Thermotop (Eppendorf, Hamburg, Germany). The resulting beads were immediately chilled on ice, the supernatant was removed and washed with 1 mL of WTA wash buffer [10 mM Tris–HCl (pH 8.0), 50 mM NaCl, 1 mM EDTA, and 0.05% Tween-20], 200 μL of BD Rhapsody lysis buffer, once with 1 mL of WTA wash buffer, twice with 500 μL of WTA wash buffer, and resuspended with 200 μL of Beads resuspension buffer (BD Biosciences). Exonuclease I-treated BD Rhapsody beads were subjected to the TAS-Seq workflow for cDNA and/or hashtag library amplification [29]. Sequencing libraries of cDNA were prepared using the NEBNext Ultra II FS DNA Library Prep Kit for Illumina, and hashtag libraries were prepared by PCR. The size distribution of the amplified products was analyzed using a MultiNA system (Shimadzu). The resulting and barcoded hashtag libraries were quantified using the KAPA Library Quantification Kit. The sequence was performed using a NovaSeq S4 200 cycle kit v1.5 and a NovaSeq 6000 sequencer (Illumina) according to the manufacturer’s instructions (read 1 67 bp, read 2 155 bp). A 12% phiX spike-in was used for the sequencing. Raw sequencing data from this experiment were deposited in the NCBI GEO, http://www.ncbi.nlm.nih.gov/geo; accession number: GSE249285).

### Time-series scRNA-seq library preparation of BLM-induced murine pulmonary fibrosis

C57BL/6 J female mice were administered a single dose of BLM sulfate (2 mg/kg dissolved in 50 μL of sterile saline solution; Nippon Kayaku) by oropharyngeal aspiration under deep anesthesia, as described previously [20]. Murine lungs were recovered on days 0, 3, 7, 10, 14, and 21 after BLM administration. The lungs were cut into small pieces with a razor blade and digested enzymatically using 0.25 mg/mL Liberase TM (Roche) and 20 kU/mL DNase I (Sigma-Aldrich) for 60 min at 37 °C. Cell suspensions were sequentially agitated with 18G and 21G needles at 20-min intervals. Erythrocytes and dead cells were removed using 25%/65% Percoll (GE Healthcare) density separation. The resulting cells were stained with Calcein AM for 5 min at 37 °C, immediately chilled on ice, and filtered through a 30 µm filter (Miltenyi Biotec). Approximately 10,000 cells were loaded onto BD Rhapsody cartridges. cDNA amplification and sequencing library construction were performed using the TAS-Seq protocol [29] using half of the resulting Rhapsody beads. The sequencing was performed using a NovaSeq S2 200 cycle kit and NovaSeq 6000 sequencer (Illumina) according to the manufacturer’s instructions (100 bp ends). A 20% phiX spike-in was used for the sequencing. Raw sequencing data from the experiment were deposited in the NCBI GEO, http://www.ncbi.nlm.nih.gov/geo; accession number: GSE201698).

### Preprocessing of scRNA-seq data

Pair-ended FASTQ files (R1: cell barcode reads, R2: RNA reads) were processed as follows: Adapter trimming of sequencing data was performed using cutadapt 4.1 [18]. Filtered cell barcode and cDNA reads were annotated and mapped to the reference genome (build GRCm39 release-107) using STARsolo (2.7.10a) and known BD Rhapsody cell barcodes using the following parameters:–clipAdapterType CellRanger4 –outSAMmultNmax 1 –outFilterScoreMinOverLread 0 –outFilterMatchNminOverLread 0 –outFilterMultimapScoreRange 0 –seedSearchStartLmax 30 –soloType CB_UMI_Complex –soloUMIdedup NoDedup –soloMultiMappers Rescue –soloFeatures Gene GeneFull –soloCBmatchWLtype EditDist_2 –soloUMIlen 8 –soloAdapterSequence NNNNNNNNNGTGANNNNNNNNNGACA –soloCBposition 2_0_2_8 2_13_2_21 3_1_3_9 –soloUMIposition 3_10_3_17. We did not perform UMI compression because the BD Rhapsody UMI length (eight-base and directly attached polyT) could not avoid UMI collision (a minimum 10-base is required) [30]. The resulting non-UMI count data were converted into gene × cell matrix files, and the inflection threshold of the barcode rank plot was detected using the DropletUtils package in R version 4.2.2 Patched (2022–11-10 r83330). Valid cell barcodes and backgrounds from inflection threshold × 0.9 to inflection threshold × 1.1 were separated using the Dropkick software package. To build the STAR index, small RNA annotations were filtered from the gencode.vM30 gtf file.

### Analysis of time-series scRNA-seq data of lung organoid and BLM-injured lungs

Single cells were clustered in each dataset using Seurat v4.0.4 [31] in R (version 4.1.3). Expression data were normalized using the normalizeData function (scale factor = 1,000,000) and scaled using the ScaleData function (read counts of each cell were regressed out as a confounding factor) in Seurat v4.0.4. Highly variable genes in each dataset were identified using the FindVariableFeatures function of Seurat v4.0.4. Thereafter, principal component analysis (PCA) against the identified highly variable genes and projection of PCA onto the entire data set were performed using RunPCA (number of calculated principal components (PCs) was 100) and ProjectPCA functions in Seurat v4.0.4. The enrichment of each PC was calculated using the jackstraw function (num. replicate = 100), and PCs that were statistically significantly enriched (*p* ≤ 0.05) were selected for clustering and dimensional reduction analysis. Cell clustering was performed using the FindNeighbors and FindClusters functions (resolution = 2.5) in Seurat v4.0.4 against the significant PCs, and dimensional reduction was performed using the Python wrapper of FIt-SNE [32] v1.1.1, using the reticulate package (https://github.com/rstudio/reticulate) in R 4.1.3. Statistically significant marker genes of each identified cluster were identified using the parallelized FindAllMarkers function in Seurat v4.0.4 (test.use = “wilcox,” only.pos = TRUE, min.pct = 0.1, adjusted *p* value with Bonferroni’s correction ≤ 0.05). Each identified cluster was manually annotated using previously reported marker genes as cell subset-defining marker genes, and lineage marker double-positive cells were annotated as doublets. We subclustered the cell subsets and removed contaminating subpopulations from the dataset. Cell–cell interaction analysis of the lung organoid scRNA-seq data was performed using the CellChat 1.0.0 package, considering population size (population.size = TRUE and raw.use = TRUE in computeCommunProb function) [33]. Statistically significant interactions and pathways were selected by computeCommunProbPathway and aggregateNet functions (thresh = 0.01).

#### Ephrin A3 and Ephrin A4 stimulation experiment

To assess the functional effects of ephrin signaling, lung organoids were treated with 0.3 µg/mL of bleomycin (BLM) for 48 h. Following this induction, the culture medium was replaced with MTEC Plus medium supplemented with either recombinant mouse Ephrin-A3 Fc Chimera protein (R&D Systems, 7395-EA) or recombinant mouse Ephrin-A4 protein (Abcam, ab191954) at final concentrations of 0.1, 0.3, and 1.0 µg/mL. The control group was treated with an equivalent volume of DMSO. Organoids were harvested for subsequent analysis at two time points: 24- and 96-h post-stimulation. Stock solutions of both recombinant proteins were prepared in 1 × PBS and stored at − 80 °C before use.

### Reanalysis of public scRNA-seq data of IPF Cell Atlas and cultured IPF cultured cells

We reanalyzed the public IPF Cell Atlas data [5] and IPF cultured cells deposited under NCBI GEO with accession numbers GSE136831 and GSE198153. We used cell-type annotations from the original study and extracted the entire epithelial cell population to visualize the gene expression (GSE136831). For GSE198153, we reanalyzed the data using Seurat v5 (the number of used PCA dimensions was 1:50, clustering resolution = 0.4) and annotated the identified cell subsets based on ABC marker gene expression (Supplementary Fig. S2A and S2B).

### Global gene expression similarity analysis between mouse and human ABCs by SingleR

To evaluate the global gene expression similarity between mouse and human ABCs, we performed SingleR analysis [34]. First, we converted mouse genes to human genes using the org.Mm.eg.db, org.Hs.eg.db, and Orthology.eg.db packages in R 4.3.0. Next, genes from the scRNA-seq data of mouse organoid-derived airway cell subsets and human IPF-derived airway cell subsets (GSE136831 and GSE198153) were filtered based on the intersection of genes between the human-converted mouse genes and the detected genes in the human datasets. SingleR analysis (per-cell mode, de.method = “wilcox”) was performed using human datasets as a reference and mouse datasets (gene-converted) as a query. The statistical significance of the resulting SingleR score for each cell cluster was calculated using the coin package in R 4.3.0..

### Statistical analysis

For bulk RNA-seq data, the significance of DEGs in the TCC-normalized transcriptome data was calculated using the TCC package in R 3.5.1. The significance of GO term enrichment was calculated using Cytoscape 3.7.2 with the ClueGO plugin (v2.5.7) and a two-sided hypergeometric test with Benjamini–Hochberg correction. Correction for multiple comparisons was performed using the Benjamini–Hochberg method. The significance of the enrichment of PCs and marker genes of each cell cluster in the lung organoid and time-series scRNA-seq data of the BLM-induced PF murine model were calculated using the Seurat v4.0.4 package in R 4.1.3. The significance of GO term enrichment was calculated using ClusterProfiler 4.10.0 [35], with OrgDb = org.Mm.eg.db, followed by Benjamini–Hochberg correction (pvalueCutoff = 0.05, qvalueCutoff = 0.2). The significance of ligand and receptor expression between the two groups was calculated using the Mann–Whitney *U* test in R 4.1.3. Other statistical analyses were performed using one-way analysis of variance, followed by Dunnett’s multiple comparison test using the GraphPad Prism software (v9.0; GraphPad Software, CA, USA). Statistical significance was set at *p* < 0.05.

## Results

### BLM stimulation impairs formation and growth of murine lung organoids

**Fig. 1:**
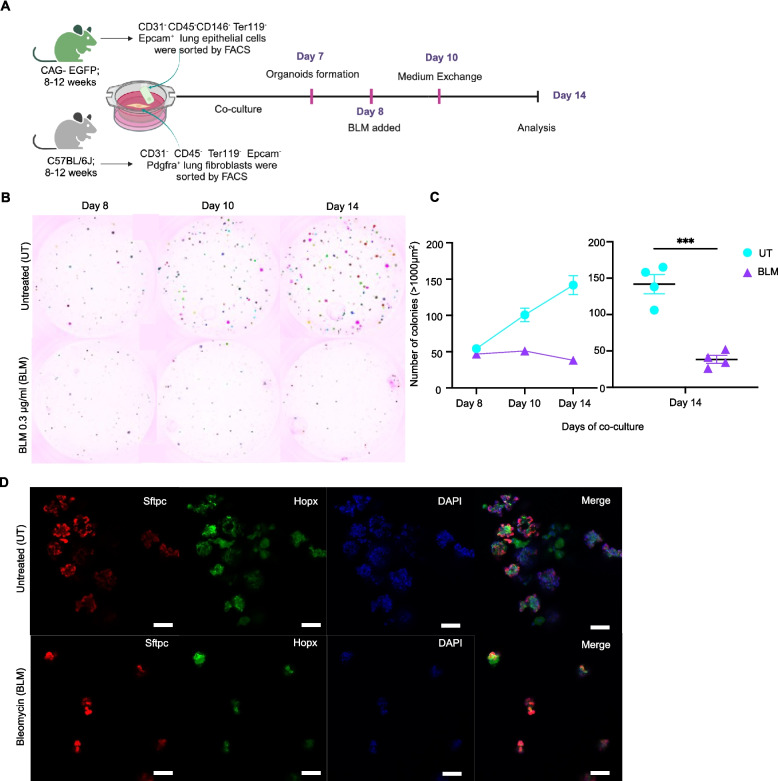
Bleomycin (BLM) stimulation impairs formation and growth of murine lung organoids. **A** Experimental scheme of lung organoid generation and BLM treatment. **B** Representative images of automatic lung organoid detection and counting from days 8 to 14 of co-culture. Each color in the upper panel represents one lung organoid inside the imaged well of the untreated (upper panel) and corresponding images of the 0.3 µg/mL BLM-treated group (lower panel). **C** Changes in lung organoid count at each time point (upper panel). Individual well data at day 14 post co-culture are also shown as a scatter plot (lower panel). Representative results from two independent experiments are shown. **C** Data are shown as mean ± standard error of the mean. (*n* = 4). *** *p* < 0.001 by two-tailed unpaired *t* test. **D** Representative images of immunofluorescence (IF) staining of lung organoids with anti-prosurfactant protein C antibody (AT2), anti-Hopx antibody (AT1), and DAPI (scale bar: 100 µm) at day 14 post co-culture. The upper panel represents lung organoids inside the imaged well of the untreated group, and the lower panel corresponds to those inside the imaged well of the 0.3 μg/mL BLM-treated group

To establish an ex vivo lung injury model using primary mouse lung cells, we stimulated murine lung organoids with BLM, a drug widely used for inducing experimental lung fibrosis in vivo. To facilitate organoid expansion tracking, we prepared organoids using EpCAM^+^ lung epithelial cells from CAG-EGFP transgenic mice and PDGFRA^+^ lung fibroblasts from wild-type mice (Fig. 1A). We found that 0.3 µg/mL BLM stimulation during days 8–10 of co-culture significantly reduced the number of lung organoids at 14 days post co-culture (Fig. 1 B and C). BLM stimulation also suppressed the increase in the number of lung organoids with a size greater than 1000 µm^2^ (Fig. 1B), and the number of lung organoids decreased in a concentration-dependent manner (Supplementary Fig. S1A and B). To analyze the morphological changes in the lung organoids in more detail, we performed immunofluorescence staining of BLM-stimulated and control lung organoids using SFTPC (an AT2 marker) and HOPX (an AT1 marker). We found that BLM-stimulated lung organoids did not form alveolar-like structures composed of spaces surrounded by AT2 and AT1 cells (Fig. 1D). These data indicate that 2-day BLM stimulation impairs organoid growth and alveolosphere formation in a murine lung-organoid model.

### Transcriptomic analysis revealed impaired lung regeneration-related gene expression in murine lung organoids following BLM stimulation

**Fig. 2:**
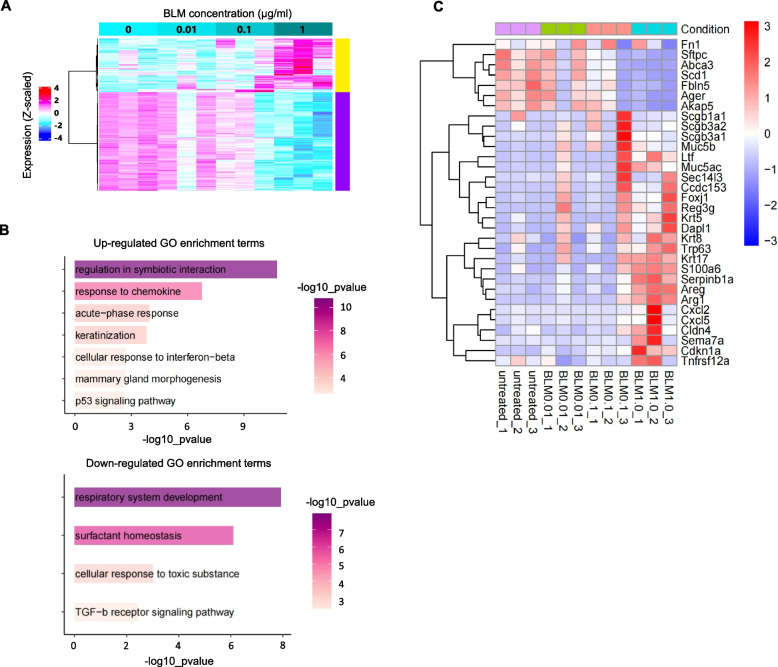
Bleomycin (BLM) stimulation impairs lung regeneration-related gene expression in murine lung organoids. **A** Heatmap representation of the expression changes of each gene in the gene modules, of which expression changes were correlated with the difference in the concentration of BLM stimulation. Upregulated and downregulated gene clusters following BLM stimulation are shown as yellow and purple clusters, respectively. **B** Gene ontology (GO) and Kyoto Encyclopedia of Genes and Genomes (KEGG) enrichment analyses for upregulated (upper panel) and downregulated gene clusters (bottom panel). The x-axis represents –log10 *p* values in each GO term, and the y-axis shows different GO terms. **C** Heatmap representation of the expression changes in key feature cell marker genes

To elucidate the characteristic changes in lung organoids induced by BLM stimulation in more detail, we performed transcriptomic analysis of the organoids 14 days after co-culture. A total of 430 DEGs (adjusted *p* < 0.05 and minimum fold chang > 2 in at least one condition compared to the control group) were identified. Clustering analysis classified these DEGs as either upregulated or downregulated in a BLM concentration-dependent manner (Fig. 2A). GO analysis of the upregulated gene cluster revealed the enrichment of genes associated with the chemokine response, acute phase response, and p53 pathway (Fig. 2B, upper panel). Conversely, GO analysis of the downregulated gene cluster revealed significant enrichment of terms related to respiratory development and surfactant homeostasis (Fig. 2B, bottom panel). In addition, the upregulated cluster included genes related to airway epithelium (*Scgb1a1*, *Muc5b*, *Krt5*, and *Trp63*), cellular senescence (*Cdkn1a*), regeneration and inflammation (*Areg*, *Arg1*, and *Serpinb1a*), and neutrophil migration (*Cxcl5*)-related genes (Fig. 2C). The downregulated gene clusters included AT2 (*Sftpc*, *Abca3*, and *Scd1*) and AT1 (*Ager*, *Fbln5*, and *Akap5*) [36] (Fig. 2C). These data indicate that BLM stimulation induced the downregulation of AEC- and lung regeneration-related genes and the upregulation of airway, inflammation, and senescence-related genes in murine lung organoids.

#### Single-cell profiling of lung organoids reveals IPF-associated aberrant basaloid cells with cross-species relevance

**Fig. 3:**
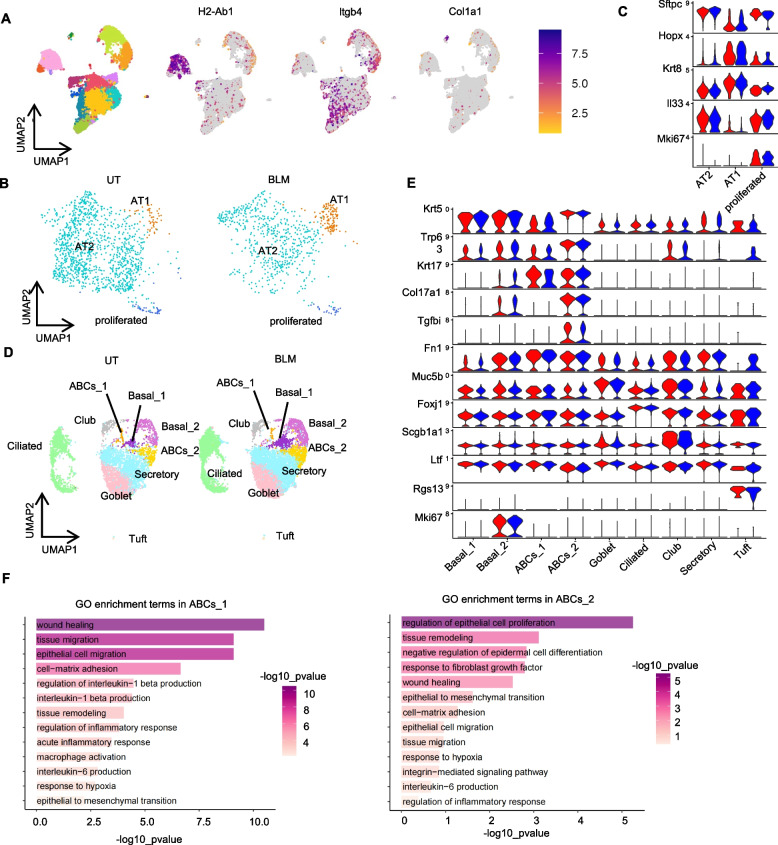
Single-cell RNA-sequencing revealed existence of aberrant basaloid cell subsets in murine lung organoids. **A** Cell clustering analysis of lung organoids visualized using a two-dimensional Uniform Manifold Approximation and Projection (UMAP) plot (left panel). Feature plots showing the expression of alveolar epithelial cell (AEC) and fibroblast marker genes in these three populations. **B** UMAP Dimplot shows the subclustering analysis of AEC subsets split by untreated (UT) (left) and BLM-stimulated (BLM) (right) with annotated cell type. **C** Violin plot indicating the expression of each cell marker gene of the AEC subsets split by UT (red) and BLM (blue). **D** UMAP Dimplot showing the subclustering analysis of AEC subsets split by UT (left) and BLM (right) with annotated cell type. **E** Violin plot indicating the expression of each cell marker gene of the AEC subsets split by UT (red) and BLM (blue). **F** GO enrichment analyses for differentially expressed genes (DEGs) in ABCs_1 (left panel) and ABCs_2 (right panel). The x-axis represents –log10 *p* values for each GO term, and the y-axis represents different GO terms

To comprehensively understand the cellular composition of our lung-organoid model, we performed scRNA-seq analysis on untreated and 0.3 μg/mL BLM-treated lung organoids on day 14 post co-culture. Among the 15,522 detected cells, AECs, fibroblasts, and airway epithelial cells were identified based on the expression of the following lineage marker genes: *H2*-*Ab1* for AECs, *Col1a1* for fibroblasts, and *Itgb4* for airway epithelial cells (Fig. 3A) [37]. Subclustering analysis of AECs identified three subsets: *Sftpc*^+^ AT2, *Hopx*^+^ AT1, and *Mki67*^+^ proliferating AECs (Fig. 3 B and C). AT1 cells also expressed *Krt8*, an intermediate differentiation marker between AT2 and AT1 [38] cells (Fig. 3C). Subcluster analysis of airway epithelial cells identified nine cell clusters: *Trp63*^+^ and *Krt5*^+^ Basal cells (Basal_1), *Mki67*^+^ Cycling basal cells (Basal_2), *Muc5b*^+^ goblet cells, *Foxj1*^+^ ciliated cells, *Scgb1a1*^+^ club cells, *Ltf* ^+^ secretory cells, and Rgs13^+^ Tuft cells (Fig. 3 D and E). We identified two IPF-related ABC clusters (*Muc5b*^−^* Scgb1a1*^−^* Krt17*^+^ *Fn1*^+^*Krt5*^*low*^ ABCs_1 and *Muc5b*^−^* Scgb1a1*^−^ *Krt17*^+^*Fn1*^+^*Krt5*^+^ABCs_2) in our murine organoid model (Fig. 3 D and E). The *Krt17*^+^ *Fn1*^+^*Krt5*^*low*^ ABCs_1 subset exists in the lungs of patients with IPF, where it specifically localizes around fibroblastic foci [5]. To further validate the similarity between human ABCs and ABC-like cells in our lung-organoid model, we performed SingleR analysis [34] using scRNA-seq data of human airway subsets (IPF cell atlas (ABC_1), GSE136831 [5]) and in vitro cultured basal cells from IPF lungs (ABC_1 and ABC_2), GSE198153 [39]) as a reference (Supplementary Fig. S2A-B). We found that ABCs_1 and ABCs_2s in our lung-organoid model showed the highest SingleR score of human ABC_1 and ABC_2 with statistical significance (Supplementary Fig. S2C, Supplementary Table S1). Because SingleR scores reflect global gene expression similarity [34], the data indicated that our organoid model could induce ABC-like cells with transcriptional similarity to human lung ABCs. In contrast, time-series scRNA-seq analysis of BLM-injured murine lungs revealed that ABCs_1 and ABCs_2 were not detected in fibrotic lungs in vivo (Supplementary Fig. S3A–C). Next, we characterized these ABC subsets based on GO analysis of their marker genes. We found that fibrosis-associated GO terms, including wound healing, cell matrix adhesion, and tissue remodeling, were enriched in both ABCs_1 and ABCs_2, further supporting the association of these ABCs with fibrotic responses (Fig. 3F). These cell subsets were detected in both control and BLM-treated organoids (Fig. 3B–D), indicating that ABC induction may not depend on BLM stimulation. These data suggest that our lung-organoid model generates ABC-like cell subsets that are transcriptionally comparable to ABCs in both primary IPF lungs and cultured IPF lung-derived ABCs. The absence of these cells in conventional in vivo BLM-induced murine fibrosis models highlights the distinct advantage of our murine ex vivo system for studying the biology of these ABC-like subsets.

### BLM stimulation induces gene expression changes in AT2 cells in murine lung organoids associated with in vivo PF model

We further investigated which cell subsets were affected by BLM treatment based on the scRNA-seq dataset of our lung organoids. We detected DEGs (adjusted *p* < 0.05, minimum percentage of expressed cells = 20%) between untreated and BLM-stimulated organoids in each cell subset and found that AT2 cells had the highest number of DEGs (65 and 31 genes were upregulated and downregulated, respectively, by BLM stimulation) (as shown in Supplementary Table S2 in Additional File 2). This observation is consistent with previous reports showing that the primary target of BLM is AT2 cells [40]. Consistent with the bulk RNA-seq data for BLM-stimulated lung organoids, GO analysis revealed enrichment in DNA repair, double-strand break repair, and cellular senescence among the genes upregulated in the BLM group (as shown in Supplementary Fig. S4A in Supplementary Figures). Next, we assessed the correlation between changes in gene expression in AT2 cells of BLM-stimulated lung organoids and those in BLM-induced lung fibrosis in vivo using single-cell RNA-seq data. The upregulated DEGs associated with the enriched GO terms in AT2 cells of BLM-stimulated lung organoids, such as *Cdkn1a*, *Trp53inp1*, *Rps27l*, *Cd81*, and *Gclc*, increased in expression from day 3 post-BLM administration in AT2 cells in vivo (as shown in Supplementary Fig. S4B in Supplementary Figures). We observed lower expression of *Blmh*, which encodes BLM dehydrogenase that degrades BLM in AT2 and AT1 cells than in the airway epithelial population in our lung organoids (as shown in Supplementary Fig. S4C in Supplementary Figures). Considering that AT2 cells are more proliferative than AT1 cells, these data partially explain why AT2 cells were the primary target of BLM-induced lung epithelial injury in the lung-organoid model. These findings suggest a partial correlation between AT2 cell responses to BLM stimulation in lung organoids and murine lungs in vivo.

### BLM stimulation enhances fibrosis-associated interactions between ABCs_2 and other cell subsets

**Fig. 4:**
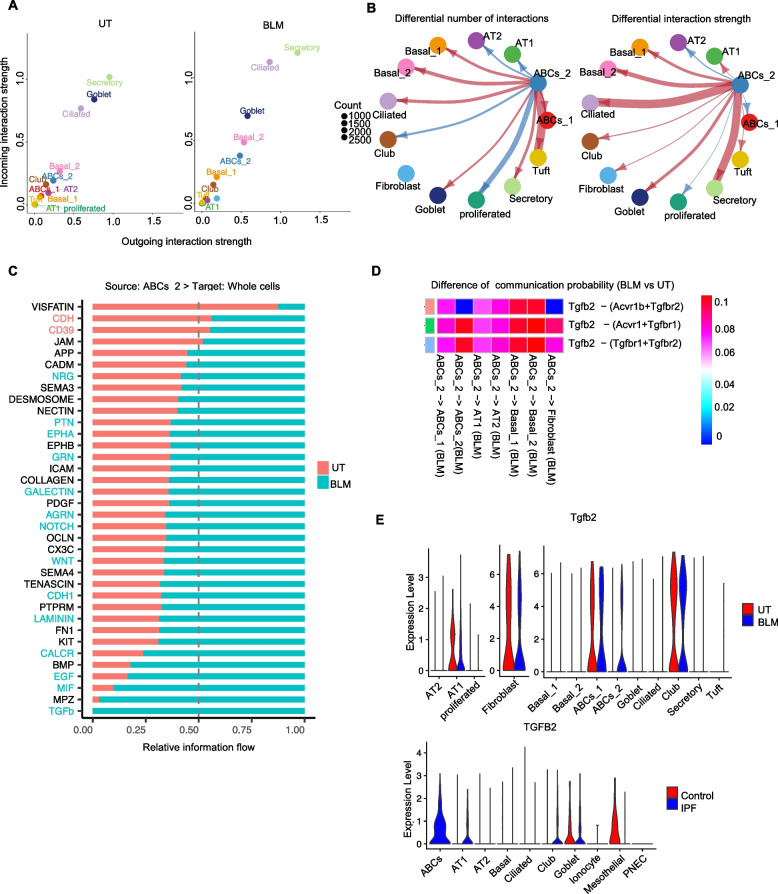
Bleomycin (BLM) stimulation enhances fibrosis-associated interactions between ABCs_2 and other cell subsets. **A** Scatter plot showing the difference in outgoing and incoming interaction strength changes between untreated (UT) (left panel) and BLM-induced lung organoids (right panel). **B** The circle plot shows the differential number of interactions from ABCs_2 to the whole cells (left panel) and the differential interaction strength (right panel). The red (or blue) edges represent increased (or decreased) signaling in the BLM dataset compared to the UT. **C** The bar plot shows the significant signaling pathways from ABCs_2 to whole cells ranked based on the difference in the overall information flow within the inferred networks between the UT and BLM groups. A paired Wilcoxon test was performed to determine the significance between UT and BLM. **D** Heatmap representation of cell-cell interaction changes at ABCs_2 of Tgfb2-mediated ligand-receptor. Upregulated and downregulated interaction strengths are colored magenta and cyan, respectively. **E** Violin plot showing ligand expression compared with untreated UT and BLM conditions in the scRNA-seq dataset of lung organoids (upper panel) at AEC, Fibroblast, AEC population, and the human IPF dataset (bottom panel)

Our scRNA-seq analysis identified ABC subsets in a BLM-stimulated lung-organoid model. To further investigate the roles of ABC subsets in response to BLM stimulation in our organoid model, we performed a cell–cell interaction analysis of the organoid scRNA-seq data using CellChat [33]. By comparing the outgoing and incoming signaling strengths of all cell subsets in untreated and BLM-treated organoids, we identified the upregulation of the outgoing and incoming interaction strength of ABCs_2 following BLM stimulation (Fig. 4 A and B). Next, we investigated the changes in the outgoing signaling pathways of ABCs_2 following BLM stimulation. We identified TGF-β signaling as the top pathway specifically induced by BLM stimulation based on information flow, which is defined as the sum of the communication probability from ABCs_2 to the other cell subsets in the inferred network (Fig. 4C). Other TGF-β and ECM-related pathways, such as the BMP, WNT, FN1, and LAMININ pathways, were also enhanced by BLM stimulation, suggesting that fibrosis-associated interactions from ABCs_2 may increase. As TGF-β is a major driver of fibroblast activation and PF development [41], we assessed fibroblast-targeting interactions in our organoid model. We found that the strength of the ABCs_2–fibroblast interaction was upregulated by BLM stimulation and that TGF-β signaling was also a top-tier contributor to this interaction (as shown in Supplementary Fig. S5A and B in Supplementary Figures). To identify which TGF-β signaling molecules possibly contribute to this interaction, we calculated the difference in communication probability through each TGF-β signaling related ligand-receptor molecule following BLM stimulation between ABCs_2 and the other cell subsets (Fig. 4D). We found that TGF-β2 and Activin A receptor type I (*Acvr1* and *Acvr1b*)- TGF-β related receptor (*Tgfbr1* and *Tgfbr2*) interactions were increased between ABCs_2 and the other cell subsets (Fig. 4D). We also found that the expression of *TGF-β2* was significantly upregulated in the BLM-stimulated ABCs_2 of the lung-organoid model, and reanalysis of the public IPF Cell Atlas data [5] revealed that *TGF*-*β2* was highly expressed in the ABCs of IPF lungs (Fig. 4E). These findings suggest that BLM stimulation enhances fibrosis-associated signaling, particularly TGF-β2 signaling, from ABCs_2 to other cell subsets in murine lung organoids.

### BLM stimulation enhances ephrin A4-EPH receptor A2 signaling from AT2 cells to ABCs_2 in lung organoids

**Fig. 5:**
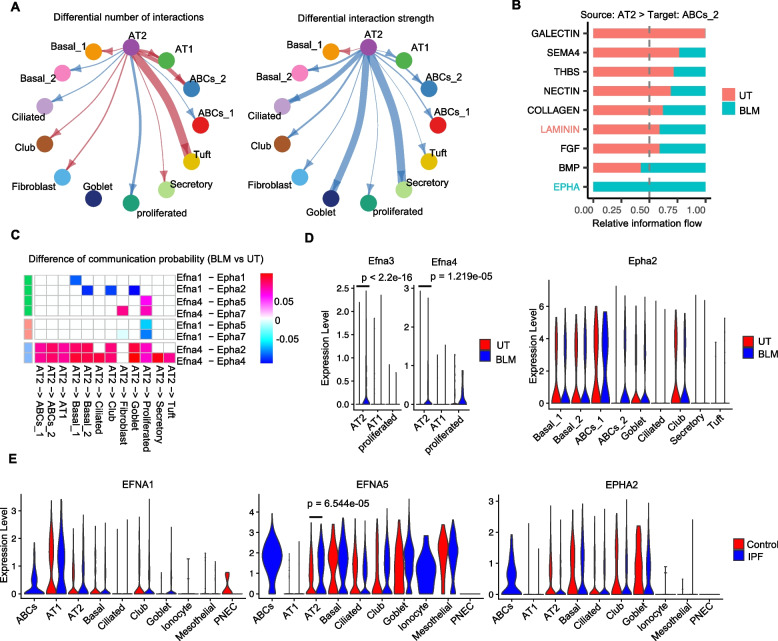
Bleomycin (BLM) enhances ephrin A4-EPH receptor A2 signaling from AT2 to ABCs_2 in lung organoids. **A** The circle plot shows the differential number of interactions between AT2 cells to other cells (left panel) and the differential interaction strength (right panel). The red (or blue) edges represent increased (or decreased) signaling in the BLM dataset compared to the UT. **B** The bar plot shows that the significant signaling pathways from AT2 to ABCs_2 were ranked based on differences in the overall information flow within the inferred networks between the UT and BLM groups. A paired Wilcoxon test was performed to determine the significance between UT and BLM. **C** Heatmap representation of cell-cell interaction changes in AT2 cells of Epha-mediated ligand-receptor. Upregulated and downregulated interaction strengths are colored magenta and cyan, respectively. **D** Violin plot showing ligand (left panel) and receptor (right panel) expression compared with untreated UT and BLM conditions in the scRNA-seq dataset of lung organoids. The expression of each ligand and receptor between UT and BLM at AT2 and ABCs_2 was correlated using the Mann–Whitney *U* test. **E** Violin plot showing ligand and receptor expression compared with the control and IPF in the scRNA-seq dataset of the human lung. The expression of each ligand and receptor between UT and BLM at AT2 was correlated using the Mann–Whitney *U* test. The exact *p* value is marked on the figure, and non-significance is marked as blank

As AT2 cell injury is the primary response to BLM stimulation, we explored the potential interaction between AT2 cells and ABCs_2. BLM stimulation increased the number of interactions between AT2 cells and ABCs_2 (Fig. 5A). Information flow analysis revealed that the ephrin-A signaling pathway was specifically upregulated by BLM stimulation (Fig. 5B). Ephrin-A signaling involves multiple family member ligands and receptors, including ephrin A1-A5 and EPH receptors [42, 43]. To identify which ephrin-A ligand/receptor might contribute to the AT2-ABCs_2 interaction upon BLM stimulation, we performed a differential communication probability analysis. We found that the interactions between ephrin A4 (*Efna4*) and EPH receptors A2/A4 (*Epha2* and *Epha4*) between AT2 cells and ABCs_2 increased following BLM stimulation (Fig. 5C). Consistent with these observations, the expression of *Efna3* (*p* < 2.2e-16) and *Efna4* (*p* = 1.219e-05) in AT2 cells and *Epha2* in ABCs_2 cells was specifically upregulated by BLM stimulation. Thus, we hypothesized that the enhancement of ephrin-A interactions between AT2 cells and ABCs_2 is associated with TGF-b2 induction in ABCs_2 by BLM stimulation. To further assess whether ephrin-A signaling is enhanced in human IPF lungs, we analyzed public scRNA-seq data from the IPF Cell Atlas dataset [5]. We found that ephrin A1/A5 *EFNA1* and *EFNA5* (*p* = *6.544e-05*) expression in AT2 cells was significantly upregulated in patients with IPF compared to that in the controls (Fig. 5E). Although EPH receptor A2 (*EPHA2*) was expressed in AT2, basal, club, and goblet cells in control lungs, it was predominantly expressed in ABCs in IPF lungs (Fig. 5E). Although both Efna3 and Efna4 were upregulated in AT2 cells upon BLM stimulation, Efna4 exhibited a higher communication probability with Epha2-expressing ABCs_2, as indicated by the difference in communication probability scores (Fig. 5C). This suggests that ephrin A4 may play a more dominant role in mediating the interaction between AT2 cells and ABCs_2.

### Ephrin A3 and A4 regulate epithelial cell proliferation, fibroblast activation, and ABC_2-related gene expression changes in BLM-stimulated organoids

**Fig. 6:**
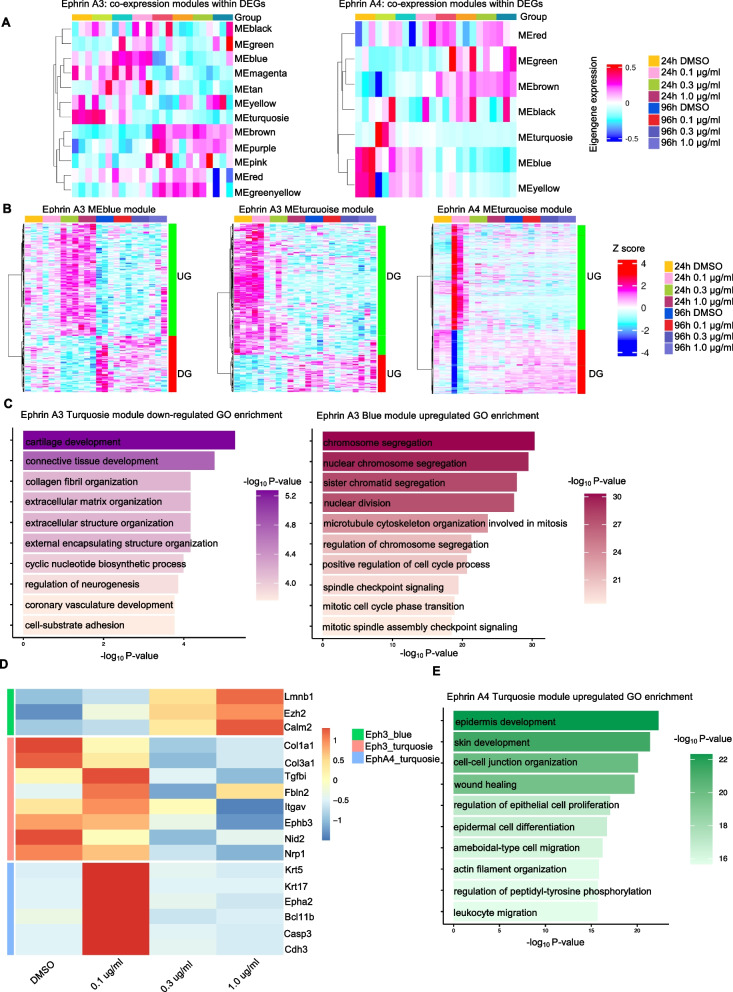
Ephrin A3 and A4 regulate epithelial cell proliferation, fibroblast activation, and ABC_2-related gene expression changes in BLM-stimulated organoids. Bulk RNA-sequencing was performed on lung organoids stimulated with recombinant Ephrin A3 or Ephrin A4 at different concentrations and time points. **A** Heatmap representation of the changes in WGCNA-identified module eigengene expression in Ephrin A3-stimulated organoids (left panel) and Ephrin A4-stimulated organoids (right panel). **B** Heatmap representation of the Z-score for relative gene expression within the Ephrin A3 MEblue, MEturquoise, and Ephrin A4 MEturquoise modules. Two differential gene expression patterns (upregulated genes [UG] and downregulated genes [DG]) were observed. **C** Gene ontology (GO) enrichment analysis for the Ephrin A3 downregulated Turquoise module, showing enrichment for terms related to ECM (e.g., cartilage development, connective tissue development, and extracellular matrix organization). **D** Heatmap showing the expression tag counts of selected representative genes associated with Ephrin A3 Blue and Ephrin A4 Turquoise modules across increasing concentrations of stimulants. The color key on the right indicates the module association of each gene. **E** Gene Ontology (GO) enrichment analysis of the Ephrin A4 upregulated Turquoise module, showing enrichment for terms associated with epithelial development, wound healing, and cell-cell junction organization. The Ephrin A3 upregulated Blue module’s GO enrichment analysis is also shown, with terms related to the cell cycle and proliferation (e.g., sister chromatid segregation, nuclear division, and microtubule cytoskeleton organization)

To investigate the functional roles of Ephrin A3 and A4 in lung organoid responses, we stimulated BLM-treated lung organoids with Ephrin A3 or A4 proteins and performed bulk RNA-seq analysis of the organoids. We identified DEGs from each Ephrin A3 stimulation experiment and the Ephrin A4 stimulation group, and weighted gene co-expression network analysis (WGCNA) identified co-expression modules within these DEGs (Fig. 6A). We found that modules MEblue and MEturquoise (Ephrin A3), and MEturquoise (Ephrin A4) showed gene expression changes (Ephrin A3: over 0.3 µg/mL, Ephrin A4: 0.1 µg/mL) in early time point (24 h after stimulation) (Fig. 6B). In module MEturquoise of the Ephrin A3 group, GO analysis of 310 downregulated genes in line with Ephrin A3 concentration revealed significant enrichment of ECM-related genes (Fig. 6C, left panel), such as *Col1a1*, *Col3a1*, and *Tgfbi* (Fig. 6D). In the MEblue module of the Ephrin A3 group, GO analysis of 205 upregulated genes in line with Ephrin A3 concentration revealed significant enrichment of terms related to the cell cycle and cell proliferation (Fig. 6C, right panel). The genes included Lmnb1, Ezh2, and Calm2, which could contribute to epithelial cell proliferation [44, 45]. In module MEturquoise of the Ephrin A4 group, GO analysis of this upregulated gene expression module revealed significant enrichment of terms related to epithelial biology, including “epidermal cell differentiation,” “wound healing,” and “regulation of epithelial cell proliferation” (Fig. 6E). Consistent with our CellChat analysis, we found that this signature included the ABC marker genes *Krt5*, *Krt17*, and Ephrin-A receptor 2 (*Epha2*) (Fig. 6D). Collectively, these results indicate distinct roles of Ephrin A3 and A4 in BLM-stimulated murine lung organoids: Ephrin A3 might enhance epithelial proliferation-associated gene expression changes and suppress fibrosis-associated gene expression changes, whereas Ephrin A4 might contribute to the induction of ABC-related gene expression changes.

## Discussion

The absence of a model that induces IPF-specific epithelial cell subsets limits our understanding of the pathophysiological roles of these epithelial cells in lung fibrosis. In this study, we established a novel ex vivo lung-organoid model that allowed the induction and activation of IPF-specific ABCs. Bulk and scRNA-seq analyses revealed that our lung injury organoid model contained ABCs with most of the lung epithelial cell subsets and partially recapitulated lung injury responses in vivo.

Our lung model demonstrated that 2 days of BLM stimulation significantly reduced the organoid size and number in a dose-dependent manner, even after stimulation ceased, mirroring the destructive effects observed in vivo following a single intratracheal BLM dose [46]. This phenomenon might be attributed to the loss of AT2 cells [12], which are critical for maintaining alveolar structures [12] because of their role as stem cells of AT2 cells and their production of essential mediators such as surfactant proteins.

Bulk and scRNA-seq analyses revealed that BLM stimulation induced significant changes in gene expression, mainly in AT2 cells, with DEGs associated with DNA repair, the p53 signaling pathway, cellular senescence, and wound healing pathways. This observation was consistent with the known mode of action of BLM, which induces DNA double-strand breaks and oxidative stress [47] and lower expression of the BLM-inactivating enzyme BLM hydrolase (*Blmh*) in AECs than in other lung cell subsets [48, 49].

A major finding of this study was that scRNA-seq analysis of lung organoids identified two ABC-like cell subsets that are a signature of fibroblastic foci in IPF lungs [39]. We demonstrated transcriptional similarity to human IPF-associated ABCs and ABC-like cells. This makes our organoid system a uniquely advantageous platform, as these ABCs were not observed in conventional in vivo murine models of BLM-induced PF. Our model generates these cells and allows us to dissect the signaling pathways that partly regulate these cell states. Cell–cell interaction analysis predicted that injured AT2 cells communicate with ABCs_2 via ephrin-A signaling. In line with this observation, Ephrin A4 stimulation suppressed ABC_2-related gene expression changes, and Ephrin A3 stimulation promoted epithelial regeneration-related and suppressed fibroblast activation-related gene expression changes, suggesting a possible contribution of Ephrin A3/A4 signaling in ABC induction, epithelial regeneration, and fibroblast activation. While BLM stimulation also induced the profibrotic cytokine TGF-β2 in ABCs, we did not observe any modification in TGF-β2 gene expression by Ephrin A3/A4 stimulation. Thus, investigating the molecular mechanisms of TGF-β2 induction in ABC_2 cells remains an important topic for future studies.

This study has a few limitations. First, the 14-day-culture period after BLM stimulation was shorter than the pathological course of the in vivo BLM-induced PF model, which includes injury (~ 7 days), fibrosis (7–28 days), and repair (from day 28 onward). This discrepancy may explain why only AT2 cells, but not other cell subsets, altered their gene expression patterns. Further analysis of the organoids for over 14 days after co-culture (e.g., 21–28 days after co-culture, which corresponds to the peak of the fibrotic responses in the in vivo BLM-induced lung fibrosis model) is required to determine whether this organoid model can reflect the patterns and phenomena of fibrotic processes. Second, the developed organoid model lacked important cell subsets that contribute to lung homeostasis, such as alveolar and interstitial macrophages [50]. Co-culture of lung organoids with these macrophages may be necessary to better identify the responses to lung injury in vivo, and thus possibly improve the model. Third, the composition of epithelial cells, particularly AECs and airway epithelial cells, may affect the response of lung organoids to BLM stimulation. As our cell–cell interaction analysis suggested communication between AECs and airway epithelial cells, the contribution of the balance of lung epithelial cell subsets to lung organoid responses should be investigated further. Fourth, functional validation experiments indicated the effect of Efna4 by stimulating BLM-injured lung organoids with Ephrin A4 protein. We found that Ephrin A4 induced ABC marker gene expression and *Epha2* expression. However, we did not perform an intervention against the Epha2 receptor; therefore, it is not clear whether the above-mentioned Ephrin A4 effect was mediated by the Epha2 receptor. In future studies, incorporating protein-level assays and neutralization approaches will be essential to establish definitive ligand–receptor engagement and downstream signaling in our organoid model.

iPSCs have been widely used in in vitro organoid models, including lung organoids, for drug screening and investigating tissue formation mechanisms [51, 52]. However, iPSC-derived lung organoids require a long generation period [15] and often contain undifferentiated epithelial cells that do not exist in the lung, with AECs in these organoids being in an immature state compared to adult AECs [15]. In addition, iPSC-derived BLM-injured organoid models do not contain ABCs [15]. In contrast, our murine organoid model was easier to handle, more cost-effective, and covered a broader range of mature lung epithelial cell subsets, including alveolar ABCs. Therefore, our model may be advantageous for drug screening in patients with human lung fibrosis.

## Conclusions

This study established a simple and rapid ex vivo lung injury organoid model that can induce IPF-specific ABCs using primary murine lung cells and BLM stimulation and found that Ephrin A4 as a contributor of the induction of ABC-related gene signatures. As various external and internal factors, such as silica/asbestos inhalation and mutations in *Sftpc*, induce lung injury, investigating lung organoid responses under these conditions may provide novel insights into lung injury, fibrosis, and repair mechanisms. Expanding our organoid approach could contribute to the development of novel drug screening platforms for lung injury and fibrosis and provide new therapeutic targets for these diseases.

## Supplementary Information

Supplementary Material 1. Supplementary Table S1. SingleR score of human ABC_1 and ABC_2 with statistical significance.

Supplementary Material 2. Supplementary Table S2. Differentially expressed genes (DEGs) of AT2 cells between untreated and BLM-stimulated lung organoids.

Supplementary Material 3.

## Data Availability

We have deposited all related data at GEO (http://www.ncbi.nlm.nih.gov/geo) with following accession number: GSE200299, GSE249285, GSE201698.

## Abbreviations

ABC: Aberrant basaloid cell
AEC: Alveolar epithelial cells
AM: Alveolar macrophage
APC: Allophycocyanin
BLM: Bleomycin
EPH: Ephrin
FBS: Fetal bovine serum
GEO: Gene Expression Omnibus
GFP: Green fluorescent protein
GO: Gene ontology
HOPX: Homeodomain-only protein X
IPF: Idiopathic pulmonary fibrosis
KEGG: Kyoto Encyclopedia of Genes and Genomes
NCBI: National Center for Biotechnology Information
PBS: Phosphate-buffered saline
PC: Principal component
PCA: Principal component analysis
PCR: Polymerase chain reaction
PF: Pulmonary fibrosis
UMAP: Uniform Manifold Approximation and Projection
UMI: Unique Molecular Identifier
WGCNA: Weighted gene co-expression network analysis
