# Supplementary material for: Ex vivo lung-organoid model for aberrant basaloid cell induction and activation
Source: Inflamm Regen. 2025 Oct 30;45:33. doi: 10.1186/s41232-025-00396-z (PMC12574287; doi:10.1186/s41232-025-00396-z)
Supplement: Supplementary file 3 — Supplementary Material 3. [file 41232_2025_396_MOESM3_ESM.pptx]

## Slide 1
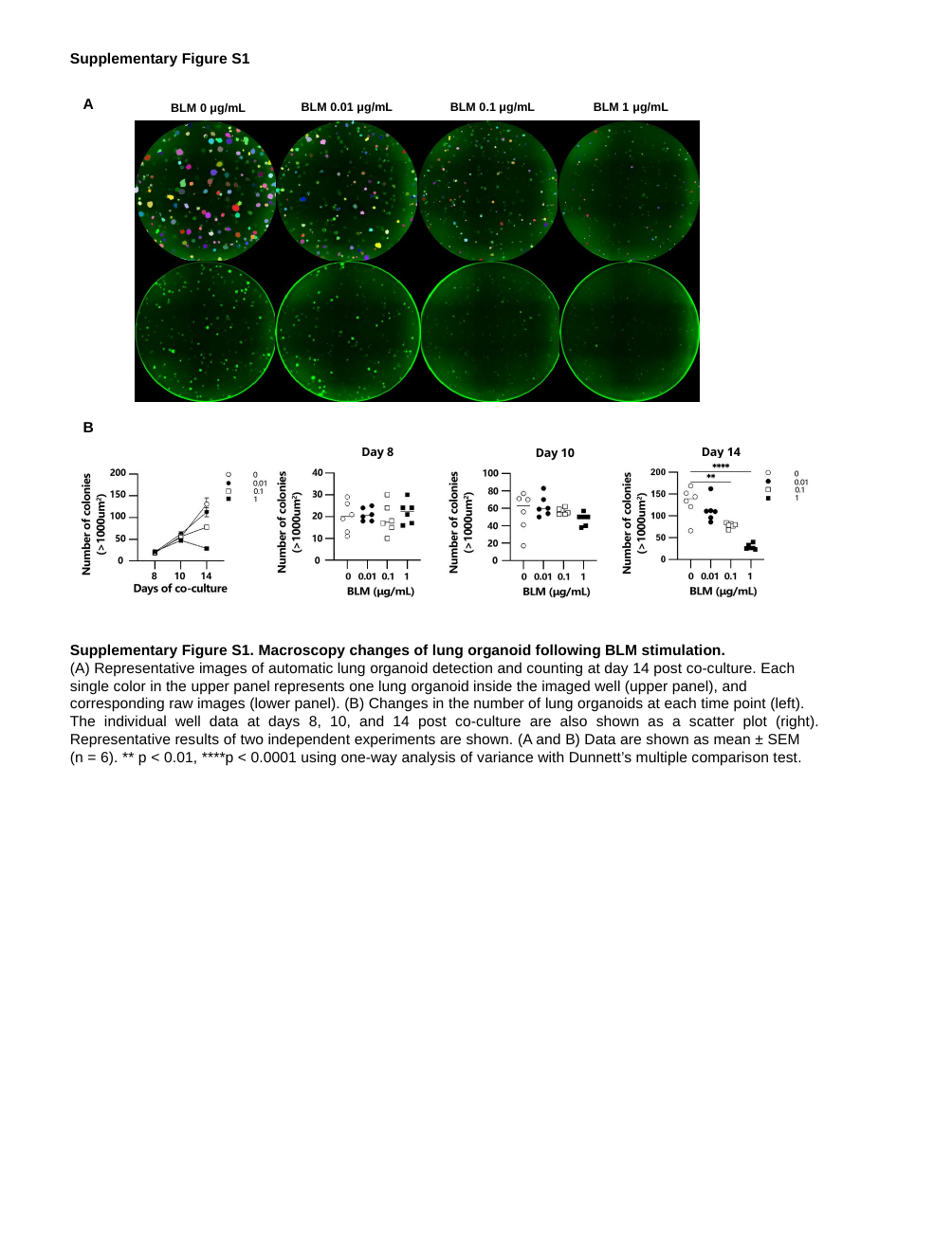

Supplementary Figure S1
A
BLM 0.01 μg/mL
BLM 0.1 μg/mL
BLM 1 μg/mL
BLM 0 μg/mL
B
Day 8
Day 14
Day 10
Supplementary Figure S1. Macroscopy changes of lung organoid following BLM stimulation.
(A) Representative images of automatic lung organoid detection and counting at day 14 post co-culture. Each
single color in the upper panel represents one lung organoid inside the imaged well (upper panel), and
corresponding raw images (lower panel). (B) Changes in the number of lung organoids at each time point (left).
The individual well data at days 8, 10, and 14 post co-culture are also shown as a scatter plot (right). Representative results of two independent experiments are shown. (A and B) Data are shown as mean ± SEM
(n = 6). ** p < 0.01, ****p < 0.0001 using one-way analysis of variance with Dunnett’s multiple comparison test.

## Slide 2
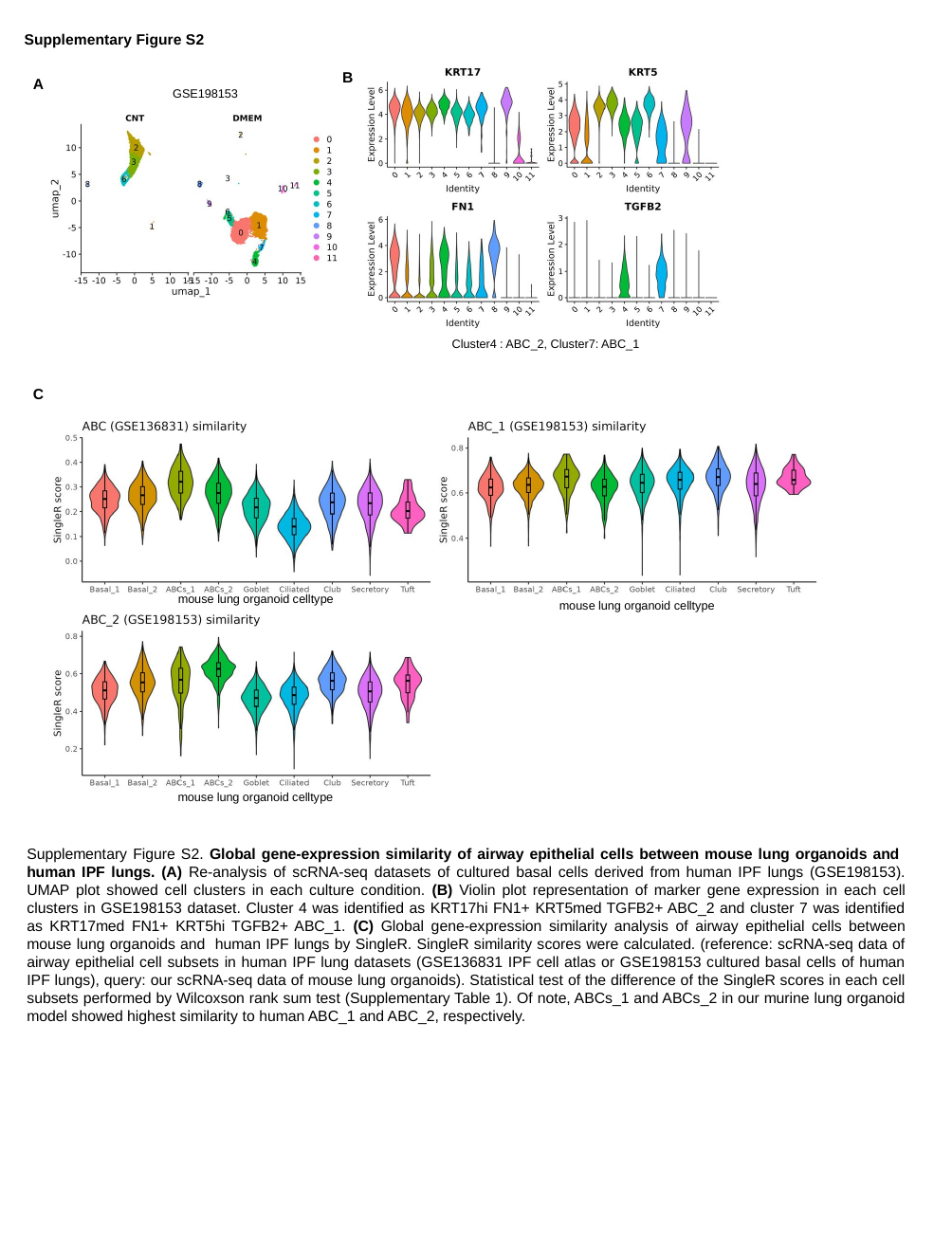

Supplementary Figure S2
B
A
GSE198153
Cluster4 : ABC_2, Cluster7: ABC_1
C
mouse lung organoid celltype
mouse lung organoid celltype
mouse lung organoid celltype
Supplementary Figure S2. Global gene-expression similarity of airway epithelial cells between mouse lung organoids and human IPF lungs. (A) Re-analysis of scRNA-seq datasets of cultured basal cells derived from human IPF lungs (GSE198153). UMAP plot showed cell clusters in each culture condition. (B) Violin plot representation of marker gene expression in each cell clusters in GSE198153 dataset. Cluster 4 was identified as KRT17hi FN1+ KRT5med TGFB2+ ABC_2 and cluster 7 was identified as KRT17med FN1+ KRT5hi TGFB2+ ABC_1. (C) Global gene-expression similarity analysis of airway epithelial cells between mouse lung organoids and human IPF lungs by SingleR. SingleR similarity scores were calculated. (reference: scRNA-seq data of airway epithelial cell subsets in human IPF lung datasets (GSE136831 IPF cell atlas or GSE198153 cultured basal cells of human IPF lungs), query: our scRNA-seq data of mouse lung organoids). Statistical test of the difference of the SingleR scores in each cell subsets performed by Wilcoxson rank sum test (Supplementary Table 1). Of note, ABCs_1 and ABCs_2 in our murine lung organoid model showed highest similarity to human ABC_1 and ABC_2, respectively.

## Slide 3
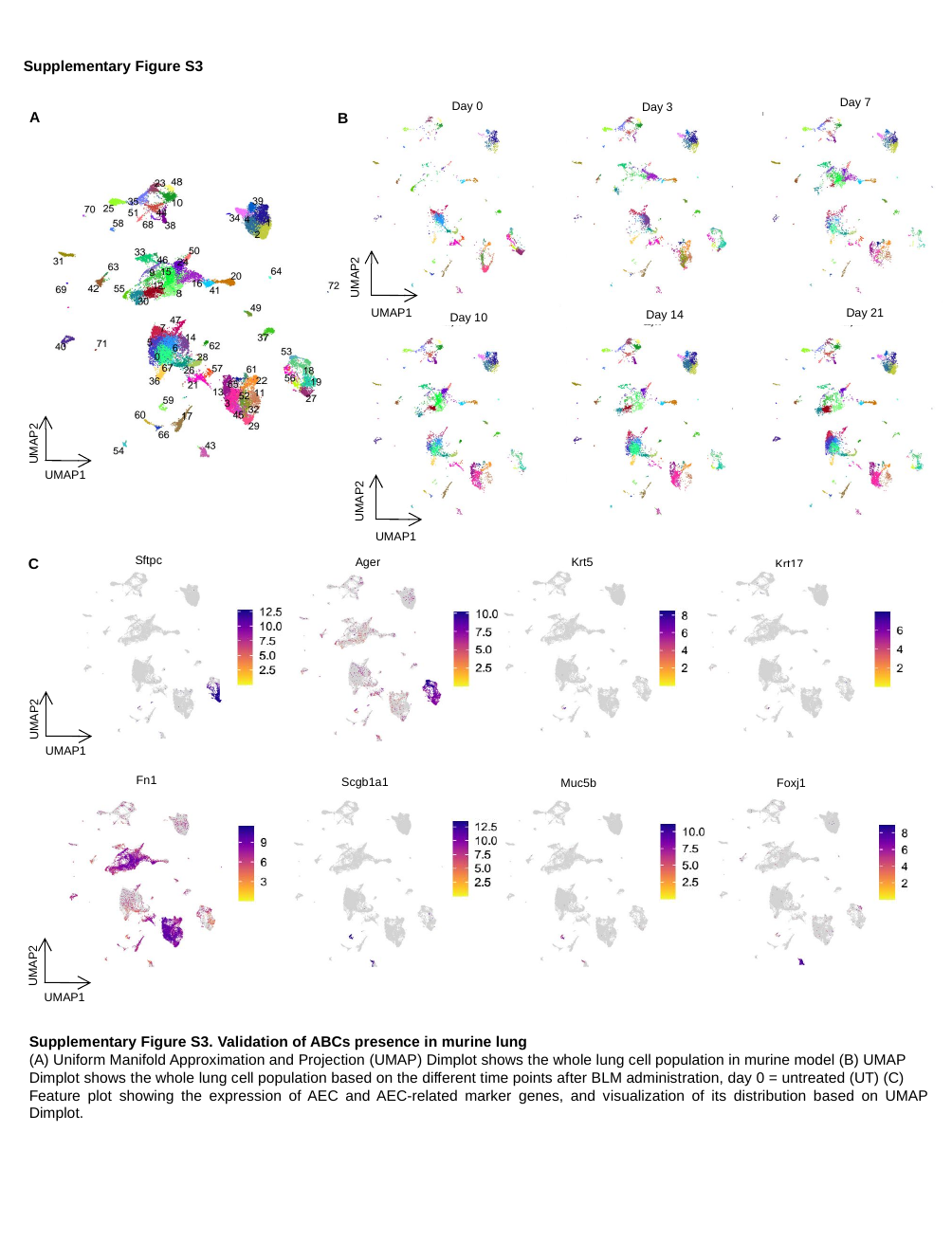

Supplementary Figure S3
Day 7
Day 0
Day 3
Day 21
Day 14
Day 10
UMAP2
UMAP1
UMAP2
UMAP1
A
B
UMAP2
UMAP1
Sftpc
Ager
Krt5
Krt17
UMAP2
UMAP1
Fn1
Scgb1a1
Muc5b
Foxj1
UMAP2
UMAP1
C
Supplementary Figure S3. Validation of ABCs presence in murine lung
(A) Uniform Manifold Approximation and Projection (UMAP) Dimplot shows the whole lung cell population in murine model (B) UMAP
Dimplot shows the whole lung cell population based on the different time points after BLM administration, day 0 = untreated (UT) (C)
Feature plot showing the expression of AEC and AEC-related marker genes, and visualization of its distribution based on UMAP Dimplot.

## Slide 4
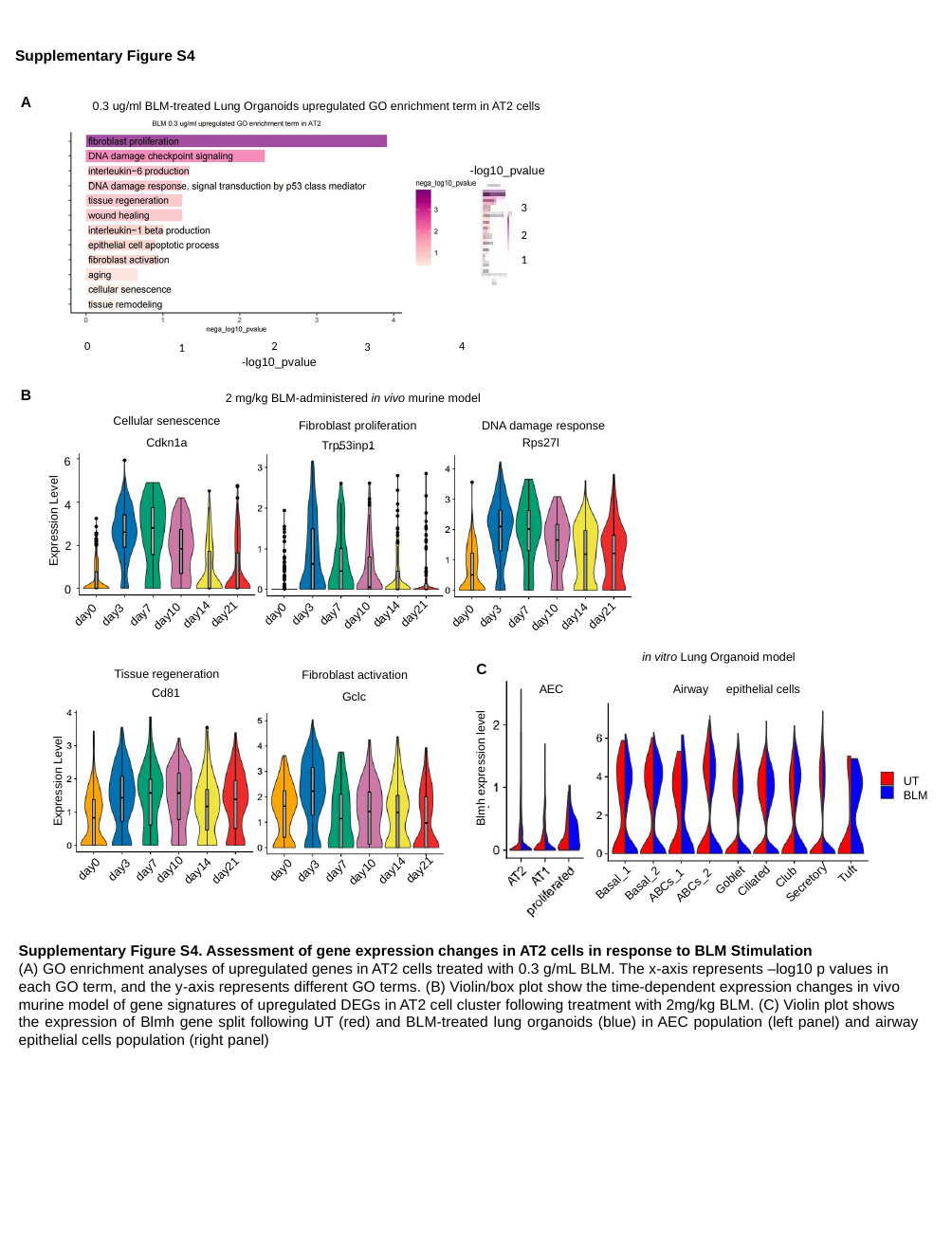

Supplementary Figure S4
A
0.3 ug/ml BLM-treated Lung Organoids upregulated GO enrichment term in AT2 cells
-log10_pvalue
3
2
1
2
4
0
3
1
-log10_pvalue
B
2 mg/kg BLM-administered in vivo murine model
Cellular senescence
Cdkn1a
6
4
Expression Level
2
0
day21
day14
day10
day0
day7
day3
DNA damage response
Fibroblast proliferation
Rps27l
Trp53inp1
day21
day14
day0
day10
day7
day21
day14
day10
day0
day7
day3
day3
in vitro Lung Organoid model
C
Tissue regeneration
Fibroblast activation
Cd81
Gclc
Expression Level
day10
day21
day0
day14
day21
day14
day7
day10
day0
day7
day3
day3
Airway　epithelial cells
UT
BLM
Club
Tuft
Basal_1
Ciliated
Basal_2
Secretory
Goblet
ABCs_2
ABCs_1
AEC
Blmh expression level

## Slide 5
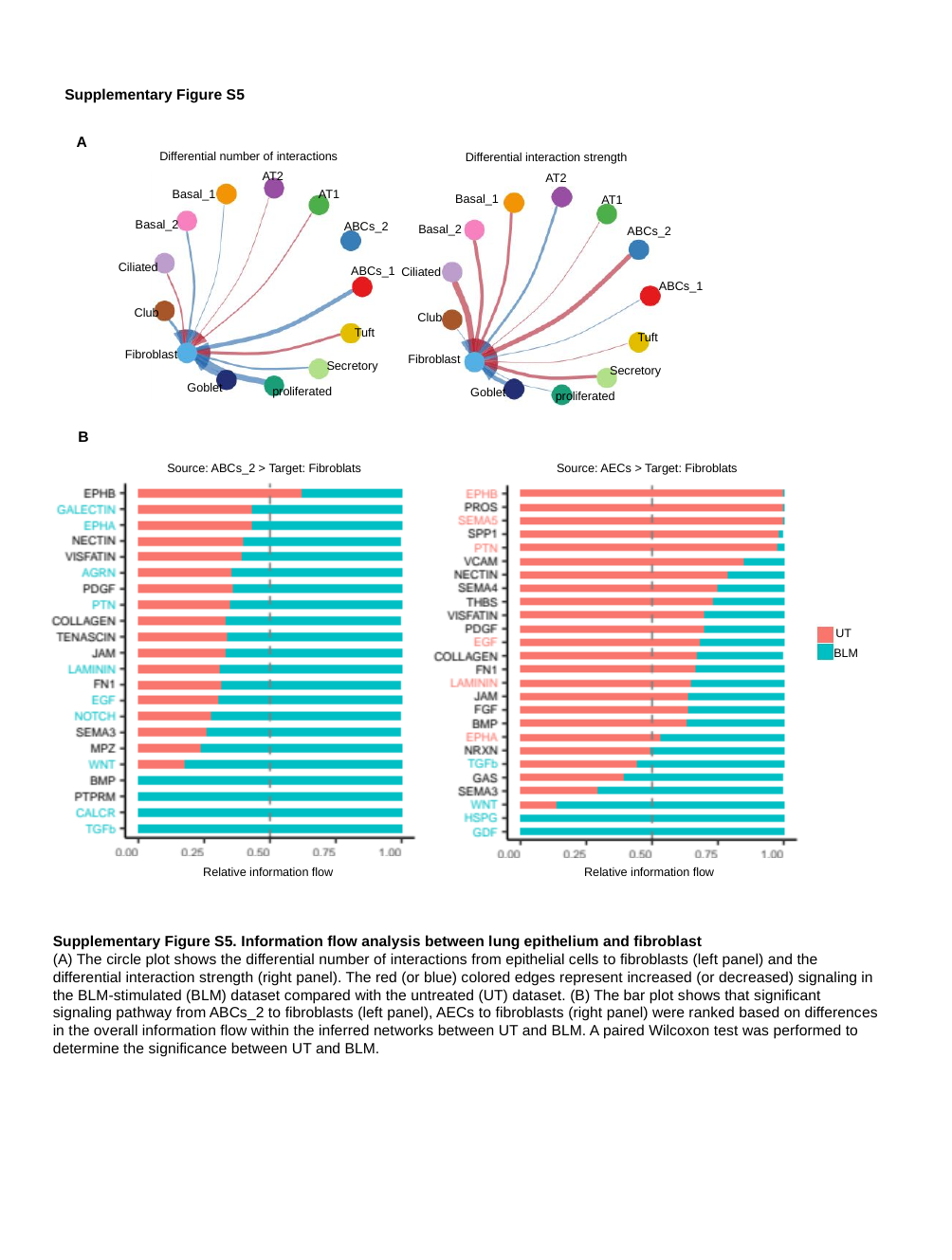

Supplementary Figure S5
A
Differential number of interactions
Differential interaction strength
AT2
AT2
Basal_1
AT1
Basal_1
AT1
Basal_2
ABCs_2
Basal_2
ABCs_2
Ciliated
ABCs_1
Ciliated
Club
Club
Tuft
Tuft
Fibroblast
Fibroblast
Secretory
Secretory
Goblet
proliferated
Goblet
proliferated
ABCs_1
B
Source: ABCs_2 > Target: Fibroblats
Source: AECs > Target: Fibroblats
UT
BLM
Relative information flow
Relative information flow
Supplementary Figure S5. Information flow analysis between lung epithelium and fibroblast
(A) The circle plot shows the differential number of interactions from epithelial cells to fibroblasts (left panel) and the
differential interaction strength (right panel). The red (or blue) colored edges represent increased (or decreased) signaling in
the BLM-stimulated (BLM) dataset compared with the untreated (UT) dataset. (B) The bar plot shows that significant
signaling pathway from ABCs_2 to fibroblasts (left panel), AECs to fibroblasts (right panel) were ranked based on differences
in the overall information flow within the inferred networks between UT and BLM. A paired Wilcoxon test was performed to
determine the significance between UT and BLM.
